# Immigration experience of Latin American working women in Alicante, Spain: an ethnographic study^1^

**DOI:** 10.1590/0104-1169.3559.2490

**Published:** 2014

**Authors:** Liliana González-Juárez, Ana Lucía Noreña-Peña, Luis Cibanal-Juan

**Affiliations:** 2 Doctoral student, Universidad de Alicante, Alicante, Spain. Professor, Escuela Nacional de Enfermería y Obstetricia, Universidad Nacional Autónoma de México, Ciudad de México, Mexico; 3 PhD, Assistant Professor, Departamento de Enfermería, Universidad de Alicante, Alicante, Spain; 4 PhD, Professor, Universidad de Alicante, Alicante, Spain

**Keywords:** Women's Health, Emigration and Immigration, Transfer of Experience (Psychology), Anthropology, Cultural

## Abstract

**OBJECTIVE:**

to describe the experience of Latin American working women regarding immigration, taking into account the expectations and conditions in which this process takes place.

**METHOD:**

ethnographic qualitative study. Data collection was performed by means of semi-structured interviews with 24 Latin American immigrant women in Spain. The information collected was triangulated through two focal groups.

**RESULTS:**

the expectations of migrant women focus on improving family living conditions. Social support is essential for their settling and to perform daily life activities. They declare they have adapted to the settlement country, although they live with stress. They perceive they have greater sexual freedom and power with their partners but keep greater responsibility in childcare, combining that with the role of working woman.

**CONCLUSIONS:**

migrant women play a key role in the survival of households, they build and create new meanings about being a woman, their understanding of life, their social and couple relationships. Such importance is shaped by their expectations and the conditions in which the migration process takes place, as well as their work integration.

## Introduction

Female migration from Latin America experienced a sustainable growth from 1960 until the year 2000, from 44.7% to 50.5%, respectively^(^ 1 ^)^. In Spain, migration flows from Latin America were characterised by a constant female predominance that increased up to 62%, during the period 1992-1996, which was reduced to 53% in the period 2002-2006^(^ 2 ^)^. This shows an increasing number of women who decided to migrate as an individual decision or as part of a family survival strategy (so that their family could have a financial means of support), a situation that is added to traditional migration for family regrouping reasons^(^ 3 ^)^.

In the last decade, women have migrated with the purpose of improving family living conditions^(^ 4 ^-^ 5 ^)^, in order to contribute to their children's future, to purchase a home for their family or to set up a business. What has been described explains how women are migrating independently, looking for work^(^ 6 ^)^, and shows a feminisation of survival in households^(^ 3 ^)^.

If we consider migration as a transition process, where people must experience adaptation situations, going from one situation to another, we must consider the aspects that have an influence on this phenomenon, framed in changes of lifestyle, health, interactions and social environments^(^ 7 ^)^. In this sense, it is important to know the conditions and personal repercussions experienced by migrant women in the destination countries, who are usually placed in working activities related to reproductive care.

The importance of reproduction care lies in the fact that these are activities related to providing food to the family, trying to keep minimum hygiene conditions and attention for the youngest and dependent family members. The fact that the woman provides this care guarantees the creation of rest time for some of the family members: usually men and the youngest, at the expense of the space the woman should enjoy^(^ 8 ^)^. This care also involves the task of looking after the health of all the family members, as well as the necessary emotional or social support^(^ 9 ^)^.

Since the incorporation of women to the labour market, the performance of reproductive care has shown a progressive trend towards commercial exploitation^(^ 10 ^)^. Thus, this care has also become a labour setting, described in the literature as a triply segregated market, with regard to gender, class and ethnic group^(^ 11 ^)^. Furthermore, they are usually cheap labour, with little free time, deficient socialisation with the work group and little opportunity for labour regulation^(^ 4 ^)^.

Therefore, the purpose of this study is to describe the experience of Latin American working women regarding immigration, taking into account the expectations and conditions in which this process takes place. Being able to investigate these matters shall allow us to analyse the repercussions and the situations that have an influence on the meaning women give to this experience.

## Method

Qualitative study performed under the theoretical approach of symbolic interactionism. This theoretical framework permits understanding how human beings give a meaning to the experiences they live through social interaction, by conditioning their behaviours and responses to everyday situations^(^ 12 ^)^. In this study, the theoretical principles permitted understanding how the experience of migrant women is shaped from the expectations they have when they migrate, as well as from interaction and the social networks they establish in the destination community.

At the methodological level, an ethnographic research was carried out, as it is a favourable method to obtain an emic perspective; showing an internal vision and increasing the understanding about the personal and working life of Latin American immigrant women. The basis of the method used as a foundation for this research was Clifford Geertz's hermeneutic anthropology^(^ 13 ^)^, conceiving culture as a fabric of facts and phenomena that have meaning. Under this concept, human behaviour is seen as symbolic actions that have meaning and value in social interactions and in the context they belong to.

This study was carried out in the city of Alicante, in the community of Virgen del Remedio, during the months of April and June 2012. Data collection was performed in two periods in order to obtain the theoretical sampling and data saturation. The women who participated in the study were immigrant women, who have been living in Spain for more than five years, with the purpose of guaranteeing a series of significant or relevant events in the personal and working life of these women during the settlement in the destination communities. The total number of participants in this study was 24 women (Table 1).

**Table 1:** - Immigrant working women in Alicante, Spain, 2012

Name	Age	Marital status	Country of origin	Migration years	Schooling	Type of activity carried out
Interview	48	Married	Bolivian	7	Secondary education	Domestic worker
Interview	41	Separated	Paraguay	5	Primary	Elderly care
Interview	45	Lives with partner	Colombia	10	Primary	Elderly care
Interview	35	Separated	Colombia	11	Primary	Domestic worker
Interview	45	Married	Colombia	5	Secondary (up to age 16)	Elderly care
Focal group 1	47	Separated	Colombia	5	Primary	Domestic worker / Elderly care
Interview	20	Lives with partner	Colombia	8	Secondary (up to age 16)	Services
Focal group 1	48	Separated	Ecuador	10	Secondary education	Domestic worker
Focal group 1	46	Married	Ecuador	7	Secondary education	Domestic worker
Focal group 1	24	Single	Ecuador	7	Secondary (up to age 16)	Domestic worker
Focal group 1	35	Single	Ecuador	7	Secondary education	Domestic worker
Focal group 1	33	Married	Ecuador	11	Primary	Domestic worker
Focal group 1	35	Married	Ecuador	7	Primary	Domestic worker
Focal group 2	42	Married	Colombia	8	Degree	Services
Focal group 2	45	Married	Peru	15	Degree	Services
Focal group 2	45	Single	Peru	1	Degree	Services
Focal group 2	42	Lives with partner	Ecuador	10	Primary	Domestic worker
Interview	40	Married	Bolivian	9	Secondary education	Domestic worker
Interview	48	Lives with partner	Bolivian	10	Secondary (up to age 16)	Services
Interview	38	Married	Argentina	10	Secondary education	Services
Interview	39	Lives with partner	Colombian	9	Secondary education	Services
Interview	32	Married	Brazil	12	Secondary education	Services
Interview	41	Single	Brazil	10	Secondary education	Domestic worker / Elderly care
Interview	40	Married	Bolivian	6	Secondary education	Elderly care

The data collection techniques were participant observation, semi-structured interview and the focal group. A semi-structured interview was conducted with 13 immigrant women. Access to the interviewed women was through the association "Fundación Alicante Acoge", whose mission is to look after the migrant population. Interviews took place at the participants' address and, when this was not possible, it was agreed to meet in a public establishment with no noise to gather the information. Interviews were audio recorded, literally transcribed and had an approximate length of 30 to 40 minutes. The interview outline was designed with open questions, enquiring about issues related to the experience of women about immigration, its repercussions and the situation they live at a working and personal level, trying to answer the purpose of the research. For example, some of the questions asked were: What were your expectations before coming to Spain?, Could you describe what situations you had to experience to adapt to this culture?, What are the repercussions you think this immigration process has brought to your life?, How do you feel your life is in this country?, among others.

In order to triangulate the information, two focal groups were formed. The first one was developed in the facilities of the University of Alicante, where seven women participated, and the second in the facilities of the "Centro Socio Educativo Unamuno", located in the community Virgen del Remedio, where four women participated. The sessions were moderated by the main researcher, with the support of the members of the research team.

The purpose of the focal groups was to collect the accounts of women regarding their personal and working experience in the destination communities and to be able to know different points of views and experience levels. The text produced by the discussion groups was recorded on tape and video, taking about one hour. Furthermore, some details that were deemed important about the development of the focal groups were registered in a field diary.

With regard to the data analysis, a manual descriptive analysis was carried out, where the first step in the analysis process was a detailed reading of the information. The principles of the reference theoretical approach guided the construction of emerging categories. Later, the data were segmented, by separately coding the focal groups and the interviews. Then, the information gathered was compared and contrasted, and significant text excerpts of the study participants' accounts were extracted. This permitted the identification of conceptual similarities between what the participant women observed and expressed. The analysis process was carried out with the support of the qualitative analysis software Atlas ti, in order to structure the emerging categories and subcategories.

At the beginning of the study, the confidentiality principles, the informed consent and the potential benefits and risks of the research were discussed with the participating women. These principles were considered during the entire research process. The participants were aware of their right to know that the conversations were being recorded and to decide to cancel their participation whenever they deemed appropriate. This study was approved by the University of Alicante, which included its ethical approval.

## Findings

From the research, a main category emerged, called integration paradoxes. This category is made up of four subcategories: migration expectations, cultural adaptation, transformation of the female role and female syncretism. Now we shall briefly give the description of the subcategories and their different thematic units.

### Integration paradoxes

#### Migration expectations

*Working and making money*; *returning and improving living conditions.* Most women came to Spain with the intention of making money, working and returning to their home countries to build a house, with the purpose of improving their family and personal living conditions and their future life in their home countries. The return to their countries is an idea that underlies the discourse of immigrant women as something that could happen sometime, but they perceive it as something distant. Anyway, the idea of returning is constantly present in their lives, as described in this account: *Eh... The expectation, well, making money, working, and returning to our country to build a good house (EN01GF1-P01).*

*Immigration for professional development. *When they considered immigrating and they took the decision, some of the women had the purpose of studying or having their children study. However, they state it was difficult to reconcile the need to work with the possibility to study. Women choose, organise, reproduce and transform meanings^(^ 12 ^)^, given that their living situations may prevent them from fulfilling their expectations. Two examples of these limiting factors are: not speaking Valencian (the regional language of the Valencian Community in Spain) and having to work to support themselves: *I did not realise that, without papers, I would not be able to study. Then I was lucky to find a job after three months and my papers were dealt with. The procedure took a year, then, during that year I obtained my papers, but I devoted to working and I did not study (INT. 11).*

*Immigration for change of life.* In their discourses, women pointed out as something important the expectation of achieving a life experience different from the one they had in their home countries. Nevertheless, their accounts reveal that the reasons that prevail in the migration decision-making are financial: *Well, maybe the reasons were financial, and well, giving myself a new possibility to improve my life, because I'm an orphan as I lost my father and mother and migration came up as a possibility to achieve my goals (INT. 04).*

*Immigration to form a family.* One of the constant expectations of women during the migration process is obtaining a family environment, providing emotional support. Very often they form a new family, with men from other nationalities. Thus, their environment and family situation is made up of multicultural families, established from immigration. As they related in the following account: *So, amid this solitude, you find (Colombian) the person who is now my partner, he is from Ecuador. And well, he had also just left a relationship in Ecuador, he has a son there and needed a woman here in Spain, to help him, who accepted that son he has and I saw no objection, to go on, then our daughter came (INT. 04).*

*Children regrouping.* An essential component for the wellbeing of a working migrant woman is the regrouping of the children. They initially travel on their own and then they promote family regrouping when they are settled in. Children usually remain under the care of grandmothers or sisters, or the fathers and older children.

During the time they are separated, women relate that they experience sadness and anguish, emotions that are reflected in their state of mind and joy for life. A factor that calmed them down was being in touch with their family through phone calls and Internet contact. They attribute importance to living the migration process together with their children and see it as an irreparable loss the fact of not being able to be with their children every day, as shown by the following interview excerpt: *What I missed the most when I arrived in Spain was that I did not have my two children. I could not do it, but without thinking I accepted coming to Spain, I couldn't, everyday it was this obsession to call my children even if it was 6 am in Colombia, every day to keep them company on the phone, to keep my daughter company when taking her bath, going to school; the same for my son, until I went to get them. I was not able to leave them there (INT. 05).*

#### Cultural adaptation

*Support for arrival.* This subcategory refers to family or friend support networks. Sometimes, settled immigrants may be the ones motivating their relatives and friends to come and look for work, or this decision is taken within families. Real chains are created between family members or friends who are in the issuing country as well as in the settlement country. This is how they receive the support for the trip, maintenance and accommodation temporarily, until they find employment, which is when the migration expectation is fulfilled.

We can see the participation of migration "companies" that provide support to the immigrant in return for payment, usually as a result of mortgaging their properties in the home country in order to obtain financial solvency for the trip and pay the initial stay expenses in the destination country. Some payments include commissions for an invitation letter, support for the trip, among others: *This contact was through a lady who helped me to obtain a ticket in a travel agency, but in return for this activity a commission was paid (INT. 08).*

*Social support for daily life.* In the performance of daily life, women value interaction with previously settled relatives, with neighbours or friends, from a Latin American or Spanish origin, or EU-migrants, in order to have support for certain daily life activities. Such support consists of childcare per hour, help to pick up children from school and in moments of sickness. According to the following account: *I think here, if you don't get or make friends, you find it impossible to live, the need for a friend here is much stronger than in your own home country (INT. 09).*

*A stranger in the home country and taste for life in the reception country.* When women have had the opportunity to return to their home country they state they feel unsettled. The links they had established with their friends and neighbours are not the same any more. Women continue to appreciate the fact that they are close to their home family. However, they say they do not feel happy in their country, which is paradoxical. They consider that taking the decision to return would be like starting life again: *Practically a stranger, not within my family, but in the neighbourhood. And in the neighbourhood I really was a stranger (EN01GF1-P03).*

Life in the settlement country is assessed as a quiet and orderly life, where you can purchase what you need to live and attain a better future. In spite of this positive assessment, this experience may also be considered stressing: *Well, life is quiet, that is the positive thing, I have to say that it is easier to purchase things" (INT. 04), "I like life here, because it's like more orderly (EN01GF1-P01).*

*Appreciating the home culture.* Women relate that migrating has allowed them to appreciate their culture, their people, and Latin American customs. They observe the migration process as a learning experience that could be useful in case they returned to their home countries: *I had to be far away from my people, my country, my culture to appreciate all I really have and that the greatest richness is in my country. In other words, it was somehow useful to come to Spain, I have discovered I have a wonderful mother, that I have an amazing country, a very beautiful culture and that it is a pity I had to leave to realise that (INT. 03)*

#### Transformation of the female role

*Personal maturity.* Women declare that the migration process has caused several changes in their life: maturity, greater responsibility and personal growth. They have learnt to appreciate life. One of the elements that has favoured these changes is interaction with people from other cultures: *I think that made me mature a lot, more as a person, that I have learnt that you have to earn things, I have learnt to appreciate what I have and what I had, so, I think it is an experience and an asset in everybody's life (INT. 10).*

*Perception of greater freedom.* Women consider that the female role with regard to exercising power and the roles assumed by each gender have changed, they have transformed given that they are immersed in another culture, for them their way to act is different, they consider they are more managerial and have greater communication in their couple relationships. The sexual freedom shown by women in the settlement country made them change their mind, for example they think they have more freedom in the way the dress and the clothes they use. In the home countries, there is still prejudice regarding these aspects, which is linked to the Latin American chauvinist culture: *Here I have felt more freedom, in the sense that I can wear such clothes, such shoes, because they are not looking at you from head to toes, because in a neighbourhood (home country), well, they all look at you. So here I feel more freedom, I walk in the street and like no one looks at me (EN01GF1-P03).*

*Role renegotiation and power relations.* Migrant women think that, since they migrated, the way in which they take decisions as a family towards life and dynamics has changed. They state they are not subject to their partner's decisions, and they use negotiation for certain situations. Nevertheless, they state they have greater responsibilities in the care for children than their partners. Greater participation by the partner is observed in home hygiene activities and less control in the time of the immigrant woman. They consider that the partner does not feel comfortable with this change in the way she is; sometimes he complains about her behaviour, compared to the behaviour she had in the home country (if they stay in the same relationship they had before migrating). The following account explains what they think about this: *I am not, let's say, submitted any more, but quieter, caring for the children, that's true, but not caring for them (partners), what time they will come, what time I have to prepare the meal, and this and that (EN02GF2-P04).*

#### Female syncretism

*Role persistence.* In some discourses, women revealed the difficulty to coordinate work, childcare and family. With regard to that, they relate scarce social support for childcare and the performance of their work activities; they must spend part of their salary to pay other women to take care of their children. This situation is reflected in the following account: *Here, you have to coordinate work and children and family, then, yes, very often for that reason I don't give my son what I should give him (INT. 08).*

## Final considerations

Migration represents for women a family subsistence strategy and a process of change, where there are difficulties to reconcile family and working life. In the destination society, they build real social support networks, the dynamics of which are essential for the development of daily life activities and childcare. As some authors mentioned^(^ 14 ^)^, family, friend or neighbour support networks make residential and work integration easier.

In the results, we can see how this group tries to improve the financial situation and find a job, a means of support, allowing them to promote their social and family development. This situation coincides with what other authors say^(^ 14 ^-^ 15 ^)^, as they point out the importance that immigrant women attach to financial security and social standing as indicators of the perceived health condition^(^ 16 ^)^. This is shown by some studies, which also mention the business field that some women explore when they migrate. This alternative may take place through two channels: being businesswomen in the destination country and/or helping their families so that they can open a source of income and business in their home country. This situation enables their professional development, their social mobility, thus reaching financial support^(^ 17 ^)^.

In this study, the role of women in household survival can be realised. This role may be assessed in different dimensions: firstly, in the performance of reproduction care, i.e., their children and home, as well as in the setting up of a new family in the settling country. The other is the one performed within family support, indirectly given through the remittances sent to the home families^(^ 17 ^)^.

The results of this study provide a glimpse of the importance these women attach to child regrouping. They state they experience sadness while their children remain in the home countries, generally under the care of grandmothers or sisters. Therefore, the mother figure is inseparable from the double dimension of caring mother and financial support at home^(^ 18 ^)^.

On the other hand, it is important to assess the material possibility offered by advances in new information technologies, communication and transport to allow social relations, which enable family units separated by migration to continue acting as a family; taking decisions and discussing the important issues that concern their members regularly. However, the lack of daily coexistence with their children for the women in this study is considered a personal unrecoverable loss, which would arouse questions on the stability of transitional families mentioned in the literature^(^ 15 ^)^.

After the migration experience, women mentioned they felt more mature and responsible in the activities they perform in the reception country^(^ 18 ^)^. They have learned to appreciate life and their personal achievement. They relate that a key element was coexistence with people from different cultures. It can be concluded that women appreciate their origin and customs. They realise that some situations cause changes that they implement in their lives, by transforming the traditional roles they had included in their daily life.

It is surprising that, in spite of the changes that migrant women perceive in their interaction with the local population, they feel more reserved and in some cases isolated^(^ 18 ^)^. This may be observed in some accounts of the women participating in the study. They report they feel like strangers in the communities where they settled, probably due to the scarce links established with the extended family, friends and neighbours. This ambiguity within the immigration experience lived by women is also described in a study performed in the Basque Country, where participants admit they would like to return to their country. Nevertheless, after going through the initial period, they agree that they do not regret having come and are thankful for everything the reception country has provided them^(^ 19 ^)^.

The population of migrant women initially reproduces the social rules, values and attitudes that are similar to those in their home country. However, over time, immigrants gradually adopt the values and certain cultural patterns in the destination country. Thus, the adaptation process makes it possible for them to appreciate their origin and culture, turning it into valuable knowledge for their life. They feel that their country's customs provide something, so they assess the elements they consider positive and those that are not, in each of the ways of living they have experienced in these places^(^ 20 ^)^.

From the perspective of migrant women, there is a greater participation of the partner in certain household activities, although they keep a greater responsibility in child and family care. The preceding is in keeping with what has been described regarding the fact that migration gives the possibility to become a negotiating agent and enter the decision-making space, which involves tensions due to the male power loss it entails^(^ 21 ^)^.

In accordance with the findings in literature, the group of women participating in this research mentioned taking decisions through negotiation with the partner and sharing childcare to a greater extent than in the home country. However, women state they have difficulties to reconcile work and family life^(^ 17 ^)^. On the other hand, they perceive they have greater freedom to talk about sexual issues with their partners and are more managerial, with greater ease to negotiate and communicate with their partners. Related to this situation, and as an element that influences the well-being level of this group, we have the scarce leisure, recreation and rest opportunities, as most leisure activities are shared with their children and family.

Culturally, Latin American women have been taught the need to be good wives, housewives, daughters and mothers, to improve the living standard of their families and to guarantee the present and future wellbeing of their children and to sacrifice to a certain extent everything related to their work, education and personal ambitions in favour of their children and their family's wellbeing^(^ 22 ^)^. The preceding is proved when women develop multiple social roles, which need to be reconciled in everyday life. Such conciliation confronts them with a daily challenge, to the extent that the association of various action fronts involves substantial wear, caused by the intense routine of the activities imposed^(^ 23 ^)^.

## Conclusions

Migrant women play a key role in the survival of households, they build and create new meanings about being a woman, their understanding of life, their social and couple relationships. Such importance is shaped by their expectations and the conditions in which the migration process takes place. Migration as a process provides women with new negotiation tools with their partners. Nevertheless, the so-called female syncretism or so-called second gender still prevails: "being for another". Their experiences are marked by the support, the social networks they have and the scarce professional fulfilment opportunities they achieve in the destination country. Finally, it may be pointed out that all factors and elements that determine the experience of the immigrant women must be assessed in further depth from the healthcare perspective. This would allow us to know the needs and care this population requires, where the emotional burden the immigrant woman withstands is considered, as well as the consequences the decision to migrate entails with regard to their life and health.
